# Benign breast disease with malignant imaging features: a case report

**DOI:** 10.1186/s13256-023-03931-z

**Published:** 2023-05-16

**Authors:** Yuanyuan Zhong, Yiqin Cheng, Ling Chen, Yulian Yin

**Affiliations:** 1 Department of Breast Surgery, Longhua Hospital Affiliated to Shanghai University of Traditional Chinese Medicine, Shanghai, 200032 China; 2 Department of Pathology, Longhua Hospital Affiliated to Shanghai University of Traditional Chinese Medicine, Shanghai, 200032 China

**Keywords:** Diabetic mastopathy, Breast cancer, Pathological diagnosis, Surgery

## Abstract

**Background:**

Diabetic mastopathy is a rare breast condition that occurs in women with poorly controlled diabetes and is characterized by hardening of the breast tissue. The purpose of this case report is to provide an overview of the clinical characteristics and therapeutic principles of this rare disease to support front-line physicians in their crucial activity of case identification.

**Case presentation:**

A 64-year-old Asian female patient with a history of type II diabetes mellitus was referred to our clinic for an evaluation of a newly discovered breast mass. The patient had been diagnosed with diabetes more than 20 years prior and was being managed with oral hypoglycemic agents. Her past medical history was otherwise unremarkable. Physical examination of the breast revealed a palpable, mobile, and firm mass measuring 6 × 4 cm in the upper quadrant of the right breast. Ultrasound images showed an uneven hypoechoic nodule, BI-RADS 4B. Mammography showed the compact and flaky nature of the two breasts and the heterogeneity of the substantive density increases. The patient’s clinical manifestations and imaging findings suggest the possibility of breast cancer. The patient opted for surgical excision of the mass. Through surgery, the mass was completely excised with negative margins. Pathological examination of the mass revealed a proliferation of fibroblastic cells, with an increased nuclear/cytoplasmic ratio, consistent with a diagnosis of diabetic mastopathy.

**Conclusions:**

This case report serves to highlight the importance of recognizing diabetic mastopathy as a possible differential diagnosis of a breast mass in patients with diabetes mellitus. In our patient, early diagnosis and treatment with lumpectomy resulted in a favorable outcome, emphasizing the importance of prompt medical and surgical management. In addition, more research is needed to mine the diagnostic marker of diabetic mastopathy and provide data related to its prognosis.

## Background

Diabetic mastopathy (DMP) is a rare complication of diabetes that affects the breasts. It is characterized by a thickening of the breast tissue, fibrosis, and calcifications. It is usually found in women over the age of 40 who have had diabetes for a long period of time. The condition is often benign, but can lead to pain and discomfort and can be a sign of underlying breast cancer. Early diagnosis and treatment can help reduce the risk of complications.

## Case presentation

**Fig. 1:**
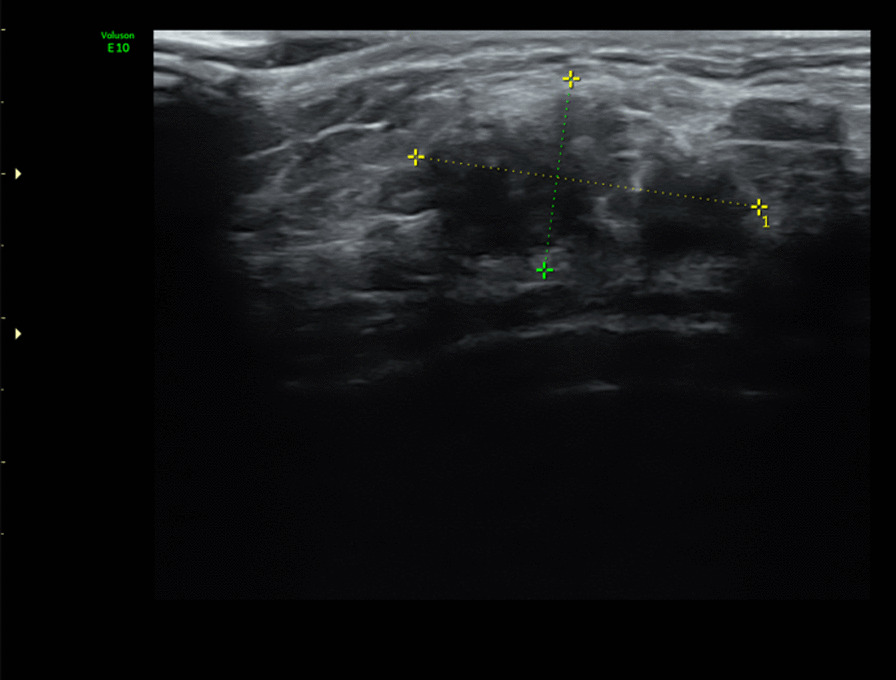
Ultrasound image of the right breast mass showing a heterogeneous hypoechoic nodule, approximately 24 × 13 mm in size, with heterogeneous internal echo, posterior echo attenuation, and poor blood supply, with an ultrasound rating of BI-RADS 4B

**Fig. 2:**
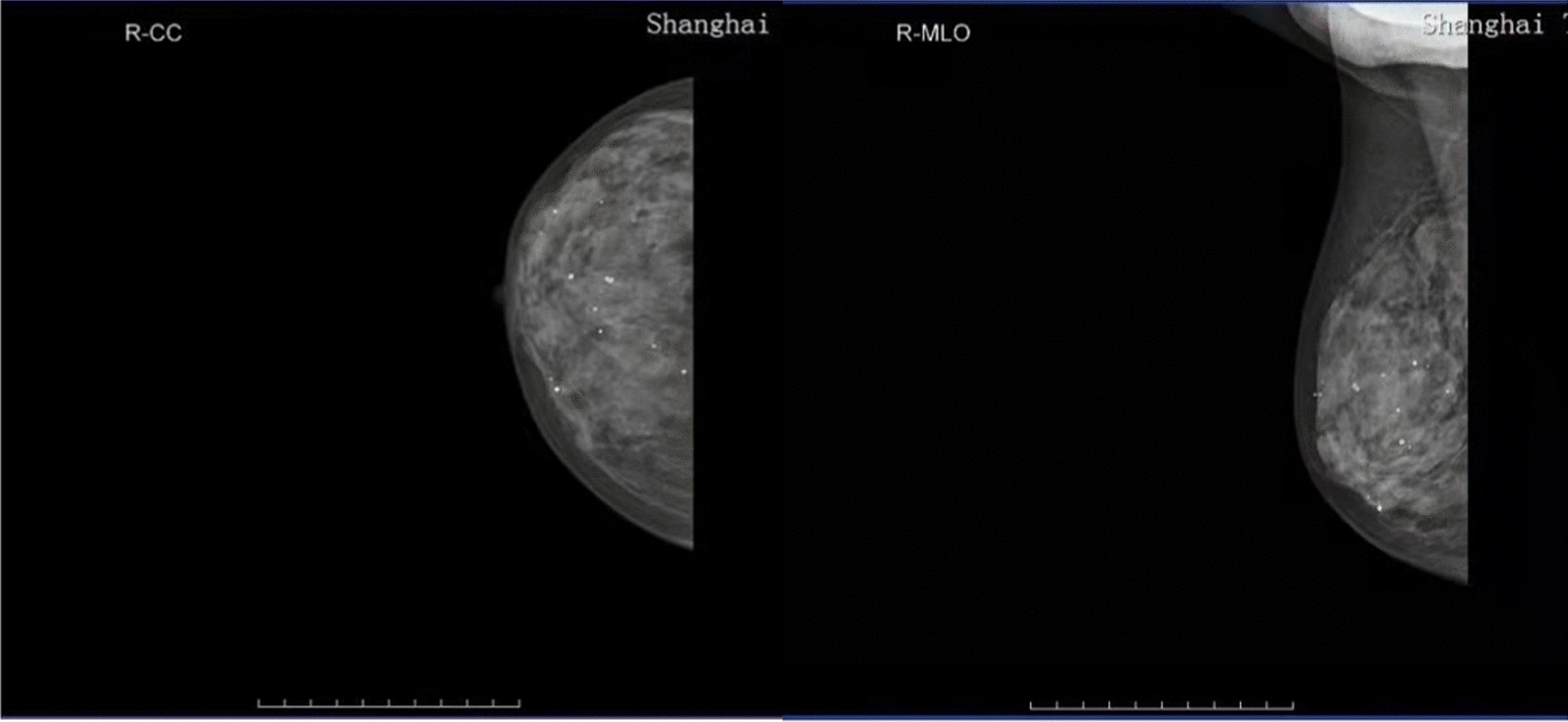
Mammographic image of breast suggesting dense, flaky nature of both breasts. Increased heterogeneity of parenchymal density. Double breast punctate calcification, BI-RADS 2

**Fig. 3:**
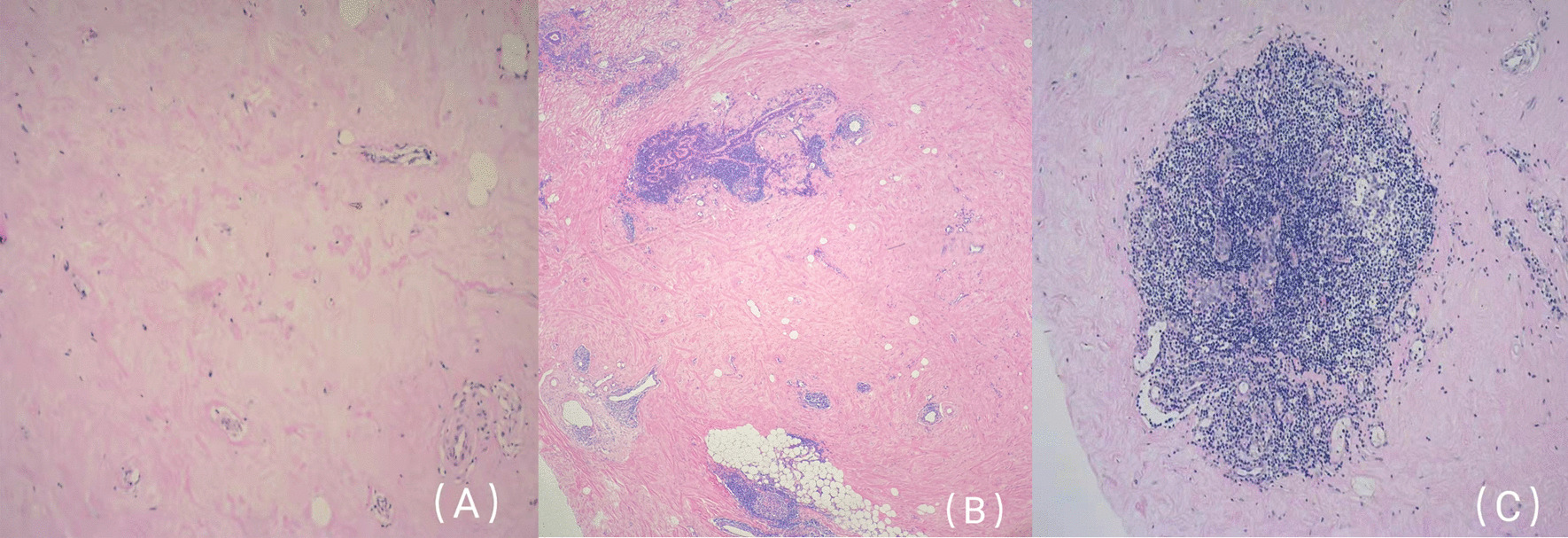
**A** Interstitial collagen; **B** more lymphocytes infiltrating around terminal duct; **C** lymphocytes in lobular

A 64-year-old Asian woman had an irregular lump about 6 × 4 cm in size on her right breast, for unknown reasons. Ultrasound images showed an uneven hypoechoic nodule in the upper quadrant of the right breast, with unclear boundary, irregular shape, backward echo attenuation, poor blood supply, and no obvious enlargement of axillary lymph nodes, BI-RADS 4B (Fig. 1). Breast mammography showed the compact and flaky nature of the two breasts, and the heterogeneity of the substantive density increases (Fig. 2), as well as double breast puncture calcification. In the course of careful medical history collection, the patient said that about 6 months ago, she felt a lump on her right breast and refused to accept any further examination or treatment. In the last 2 weeks, she decided to go to the hospital because she felt the mass had gotten harder and bigger. After the doctor’s examination and imaging examination, the attending physician informed the patient that her right breast lump might be malignant, and suggested that she immediately remove the lump to confirm the pathological diagnosis. The patient finally underwent a mass resection of the right breast, and postoperative paraffin pathology suggested DMP (also known as lymphocytic mastopathy) (Fig. 3). In retrospect, we found that the patient had type II diabetes for more than 20 years and diabetic retinopathy for more than 4 years. Her most recent glycated hemoglobin value was 8 mmol/L, with a normal range of 3.6 mmol/L to 6 mmol/L. It was obvious that she had poor glycemic control. The patient has no family history of breast cancer or DMP. A follow-up of the patient 3 months after surgery revealed no local recurrence or new breast lump.

## Discussion

Diabetic mastopathy (DMP) is a rare and specific type of mastitis that accounts for 1% of all benign breast diseases [1]. Due to its non-specific clinical and imaging manifestations and different histopathological manifestations, it is difficult to accurately diagnose before surgery, often resulting in missed diagnosis and misdiagnosis. DMP is associated with diabetes, autoimmune diseases, lymphocytic lobulitis, vasculitis, and so on. The main manifestations of this disease are lymphocytic lobulitis, ductitis, vasculitis, and dense keloid fibrillation [2]. DMP was first reported by Soler *et al*. in 1984. DMP can occur in patients with type I and type II diabetes, but it is more common in patients with type I diabetes [3]. Its pathogenesis is still unclear, and it may be related to the secondary autoimmune reaction caused by hyperglycemia. It is almost indistinguishable from breast cancer in terms of its clinical and imaging manifestations. Clinically, painless, immobile masses are often palpable. The majority of cases in ultrasound images are hypoechoic masses with unclear boundaries, irregular morphology, and posterior echo attenuation. Mammography is usually unremarkable because lesions usually appear in abnormally dense breast tissue. The typical radiological presentation is dense breast tissue, as the mass is often covered by dense breast tissue. The early pathology of the disease is lobular hyperplasia with many lymphocytes and varying numbers of plasma and mononuclear cells in the lobular, ductal, and perivascular regions. Cell infiltration was dominated by mature B cells. Epithelioid fibroblasts within the lobular interstitium are also frequently observed [4]. Historically, diabetic breast disease was primarily an overgrowth of fibrous connective tissue, usually accompanied by vasculitis and some ductal epithelial hyperplasia. The lesion was dominated by dense keloid fibrosis. There is usually little or no adipose tissue or cellular component at the site of the injury, as well as infiltration of lymphocytes, mainly B cells (inflammatory part of the lesion), around the ducts, lobules, or blood vessels.

If we come across a patient whose clinical characteristics are likely to be malignant breast masses, and she has a long history of diabetes, then we need to consider the possibility of DMP before surgery. In addition, a minimally invasive and appropriate approach must be chosen to assist in the diagnosis. There are no standardized guidelines for the treatment of DMP, and surgery has always been the focus of discussion among scholars. Some researchers have used surgery to treat this disease and found that the recurrence rate is 21.25% [5]. Another scholar believes that surgical resection should be avoided in the treatment of DMP because it can stimulate disease progression and recurrence rate after resection. Regular follow-up after the diagnosis of DMP is recommended [6]. Therefore, whether surgery should be performed and the appropriate treatment is always the focus of debate among scholars. Core needle biopsy is an alternative treatment option because it allows access to sufficient tissue to meet the needs of pathological diagnosis and avoids excessive surgery. However, there is still a lack of strong clinical evidence to support it. Therefore, this will be an interesting case for future research and deserves further attention and research.

## Conclusions

Overall, DMP is a rare disorder that requires close monitoring and aggressive management. Although the condition can be challenging to diagnose and treat, early detection and proper management can lead to a favorable outcome. Treatment should focus on controlling diabetes and managing any associated symptoms. Further research is needed to understand the underlying pathophysiological mechanisms of this disorder, as well as to develop better diagnostic and treatment strategies.

## Data Availability

All data presented in this report are included in this article.
